# The potential of Jellytoring 2.0 smart tool as a global jellyfish monitoring platform

**DOI:** 10.1002/ece3.9472

**Published:** 2022-11-01

**Authors:** Ana Ruiz‐Frau, Miguel Martin‐Abadal, Charlotte L. Jennings, Yolanda Gonzalez‐Cid, Hilmar Hinz

**Affiliations:** ^1^ Institut Mediterrani d'Estudis Avançats, IMEDEA (CSIC‐UIB) Esporles Spain; ^2^ Department of Mathematics and Computer Science, Systems Robotics and Vision group (SRV) Universitat de les Illes Balears Palma Spain

**Keywords:** artificial intelligence AI, automatic detection

## Abstract

Despite the recent recognition of jellyfish as an important component of marine ecosystems and existing concerns on their potential population increase, they are rarely monitored at the appropriate spatial and temporal scales. Traditional jellyfish monitoring techniques are costly and generally restrict the spatial–temporal resolution limiting the quantity and quality of monitoring data. We introduce Jellytoring 2.0, an automatic recognition tool for jellyfish species based on convolutional neural networks (CNN). We trained Jellytoring 2.0 to identify 15 jellyfish species with a global distribution. Our aim is to offer Jellytoring 2.0 as an open‐access tool to serve as the backbone for a system that promotes the creation of large‐scale and long‐term jellyfish monitoring data. Results reveal that Jellytoring 2.0 performed well in the identification of the 15 species with average precision values ranging between 90% and 99% for most of the species. Four of the species presented slightly lower values (75%–80%). Our system was trained on a relatively small dataset, implying that additional integration of image data will further improve the performance of the CNN. We show how the application of CNNs to image data can deliver a tool that will enable the cost‐effective collection of jellyfish data on larger spatial and temporal scales. For Jellytoring 2.0 to become a truly global automatic identification system, we ask scientists and nonscientists to actively contribute with jellyfish image data to extend the number of species it can identify.

## Graphical abstract

Jellytoring 2.0 is an automatic recognition tool for jellyfish species based on convolutional neural networks (CNN). The application of CNNs to image data can deliver a tool that enables the cost‐effective collection of jellyfish data on larger spatial and temporal scales.

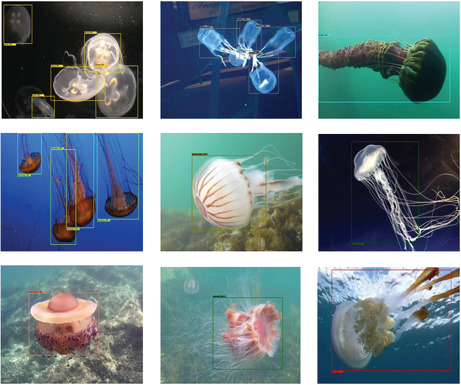

## 1 INTRODUCTION

Recent research has highlighted the importance of jellyfish in marine ecosystems (Hays et al., 2018; Lamb et al., 2019; Milisenda et al., 2014). In addition, recently recorded increases in jellyfish have been linked to global change scenarios such as high fishing pressure (Richardson et al., 2009) and global warming (Brotz et al., 2012). However, the paucity of consistent long‐term temporal and spatial data on jellyfish is such that there is considerable uncertainty over whether and why jellyfish populations might be increasing (Condon et al., 2012, 2013; Pitt et al., 2018; Sanz‐Martin et al., 2016). The logistical difficulties in sampling and monitoring these organisms in time and space are mostly responsible for this deficient level of information, hindering our understanding of jellyfish population dynamics (Lamb et al., 2019).

Generally, jellyfish are monitored by visual observations from the shore and/or boats, with data logging approaches ranging from quantitative, to presence/absence and relative abundance indices (Condon et al., 2013). Data generated in this way generally tend to have a limited spatial and temporal resolution due to the high sampling effort and labor intensity involved and their associated cost. Furthermore, human observer data collection often requires special training and is susceptible to observer bias.

Here, we explore the application of convolutional neural networks (CNNs), a type of deep learning methodology, for the identification of different species of jellyfish. The successful application of this methodology could serve as a basis for the cost‐effective acquisition of data in a continuous standardized way, with the possibility of moving toward a global scale methodology. Identification bias associated with the observer would be reduced, since networks are trained by a multitude of observers and a high volume of images which would level out any potential biases. Recent studies have successfully applied CNNs for the identification of different jellyfish species (Gauci et al., 2020; Han et al., 2021; Martin‐Abadal et al., 2020). The majority focused on the local scale (Gauci et al., 2020; Martin‐Abadal et al., 2020), while one study used jellyfish images from different geographical areas without a spatially explicit specification (Han et al., 2021). Yet, if the objective is to significantly advance and improve the quality and quantity of jellyfish data and spatial and temporal coverage, the first step is to develop a cost‐effective method that allows for efficient data collection at large scales.

Here, we demonstrate and evaluate the use of the Jellytoring deep learning neural network as the basis for a global jellyfish identification system.

## 2 MATERIALS AND METHODS

The present work extends on the technological advances previously achieved by the authors at setting the basis for an automatic jellyfish identification system. Jellytoring, a convolutional neural network, was developed and trained for the identification and quantification of three common jellyfish species in the Western Mediterranean area with successful results (Martin‐Abadal et al., 2020). The tool, however, has the potential to be further expanded to identify a wide range of jellyfish species at a global scale.

### 2.1 Convolutional neural networks

Convolutional neural networks (CNN) are a type of deep learning approach that allows the automatic extraction of information within an image. Neural networks are inspired on how the neurons in a mammalian brain respond to stimuli (Goodfellow et al., 2016). Training examples are given to the network so that it learns how to complete a task (Goodfellow et al., 2016). Here, we used underwater images containing different species of jellyfish to train a CNN to detect and classify them. Once the CNN has “learned,” it can detect and classify new jellyfish images, assigning confidence values to each detection. Finally, the species with the highest confidence value is associated with the detection.

### 2.2 Image acquisition to train the CNN

Jellytoring was originally trained to identify three common Mediterranean species, namely *Pelagia noctiluca*, *Cotylorhiza tuberculata*, and *Rhizostoma pulmo*. To expand the capabilities of Jellytoring, hereafter Jellytoring 2.0, and make its application global, underwater video recordings from a range of jellyfish species were used to obtain additional images. The search platform YouTube was used to scan for publicly available jellyfish underwater videos. Searches were performed using the keyword “jellyfish” subsequently following a snowballing technique. A total of 226 videos corresponding to 15 jellyfish species were identified. A list of the jellyfish species and their respective number of videos are detailed in Table 1. Relevant videos were downloaded, and still images were extracted from each video. On average, a frame was extracted every 5 s. To log the presence of the different jellyfish species, an annotation file was generated using LabelImg (Tzutalin, 2018). For each of the frames, a bounding box around every jellyfish occurrence was drawn and was classified according to the jellyfish species. An .xml file was generated containing the position and classification of the occurring instance within the image. Overall, a total of 3808 occurrences were recorded corresponding to 15 jellyfish species (Table 1).

**TABLE 1:** Summary of the jellyfish species included in the study, number of videos analyzed, total number of still images extracted from the videos, and geographical distribution of the different species according to WoRMS database.

Species	# videos	# files	Atl/Med	Pacific	Arctic/Baltic	Indian/China
*Aurelia aurita*	14	210	x	x	x	x
*Carybdea branchi*	3	42	x	‐	‐	‐
*Chrysaora achlyos*	14	529	‐	x	‐	‐
*Chrysaora fuscescens*	11	200	‐	x	x	‐
*Chrysaora hysoscella*	12	235	x	‐	‐	‐
*Chrysaora quinquecirrha*	4	126	x	‐	‐	x
*Cotylorhiza tuberculata*	30	449	x	‐	‐	‐
*Cyanea capillata*	16	210	x	x	x	‐
*Cyanea lamarckii*	6	123	x	‐	x	‐
*Nemopilema nomurai*	3	77	‐	x	‐	x
*Pelagia noctiluca*	58	616	x	x	‐	x
*Rhizostoma luteum*	5	138	x	‐	‐	‐
*Rhizostoma pulmo*	30	379	x	‐	‐	‐
*Stomolophus meleagris*	11	205	x	x	‐	‐
*Tamoya ohboya*	8	259	x	‐	‐	‐

Abbreviation: x, recorded in the region.

In an initial step, Jellytoring 2.0 was trained grouping all jellyfish species into one model. Performance however was not entirely satisfactory (Tables S1 and S2) as for similar‐looking species (e.g., *Rhizostoma pulmo* vs. *Rhizostoma luteum*), the network incorrectly identified them in up to 30% of the instances. To reduce the number of misidentifications and to improve the network's performance, a different approach was adopted and the CNN was trained to generate different models for different geographical areas.

**FIGURE 1:**
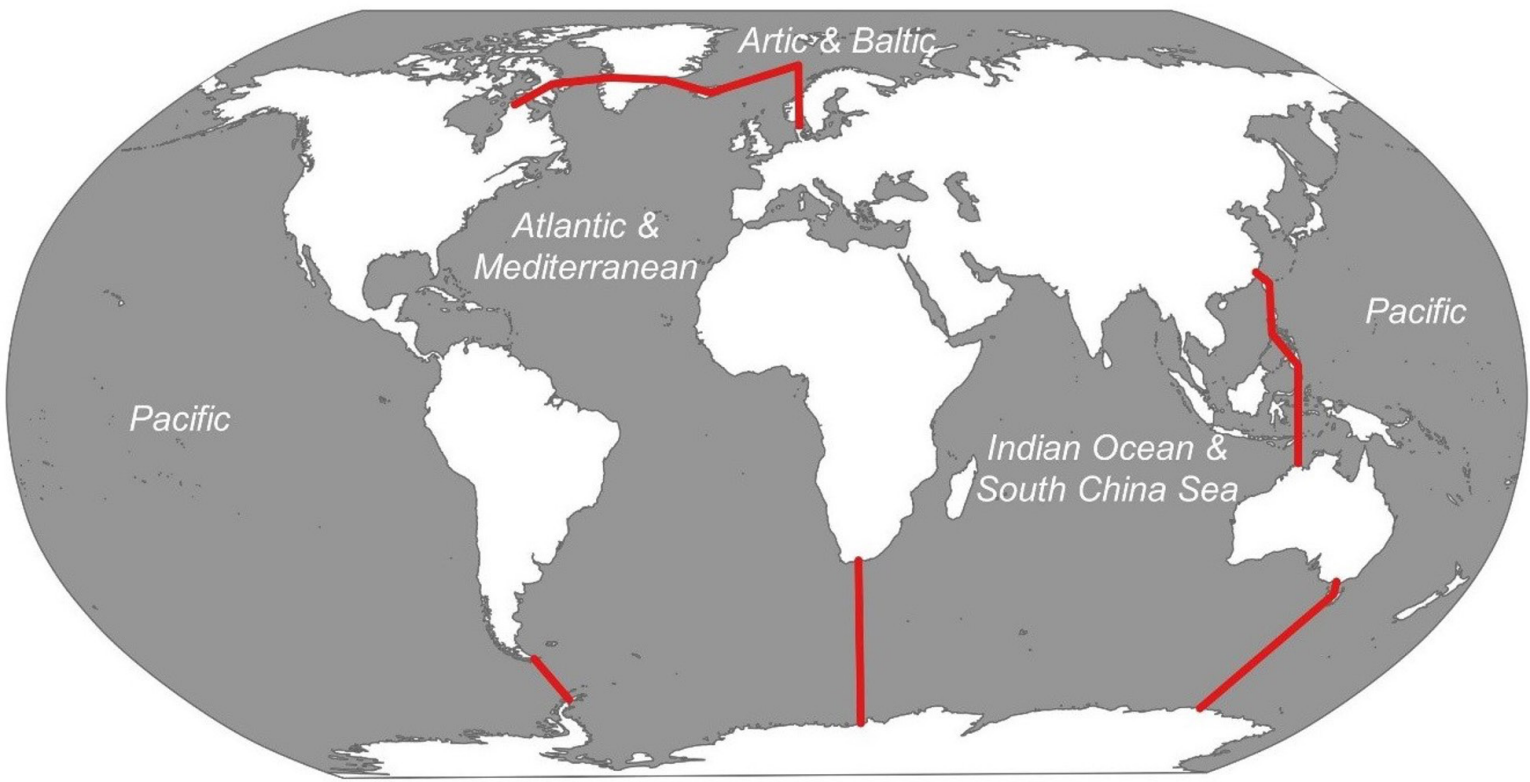
World oceans' map considered in the creation of regional neural networks to reduce error in the identification of species with similar morphology.

WoRMS database (www.marinespecies.org) was used to assess the spatial distribution of the jellyfish species, which were assigned to the different areas of the world where they had been recorded (North Pacific, South Pacific, North Atlantic, South Atlantic, Baltic Sea, Mediterranean Sea, Indian Ocean, South China Sea, Arctic Ocean, and Southern Ocean). To define the number of subareas, a multidimensional scale (MDS) plot was produced using a similarity matrix based on Euclidean distances on the presence/absence of the species for the different oceanic regions. Four distinct groupings emerged, namely the Atlantic/Mediterranean area, the Pacific area, the Arctic/Baltic area, and the Indian Ocean/South China Sea (Figure 1). Therefore, the CNN was trained to generate four different models for the four subareas. Each model was trained using data from that specific area. This approach ensured that the network could not get confused between two similarly looking species, which had been uniquely recorded in different geographical areas.

### 2.3 Framework network selection

A variety of deep learning frameworks based on CNN can be used to extract instance information from images (Dai et al., 2016; Girshick, 2015; He et al., 2016; Lin et al., 2017). Here, our objective was to select a framework with the capability of detecting and classifying a range of jellyfish species, without the need to obtain pixel‐wise segmentation of the detected instances, nor any extra feature that could slow down the process. Therefore, considering the requirements of the present application, the Faster R‐CNN framework was selected. Considering the slow movement of jellyfish, an architecture with high detection performance was deemed suitable. We selected the Faster R‐CNN‐based implementation of the Inception ResNet v2 architecture (Szegedy et al., 2016). Full details of the framework and network selection are detailed in Martin‐Abadal et al. (2020). Inception ResNet v2 is a very deep CNN with over 450 layers that can efficiently learn to identify objects within images, outputting instance bounding boxes and classifying them into one of the specified classes with a confidence percentage associated with it.

### 2.4 Jellytoring workflow

**FIGURE 2:**
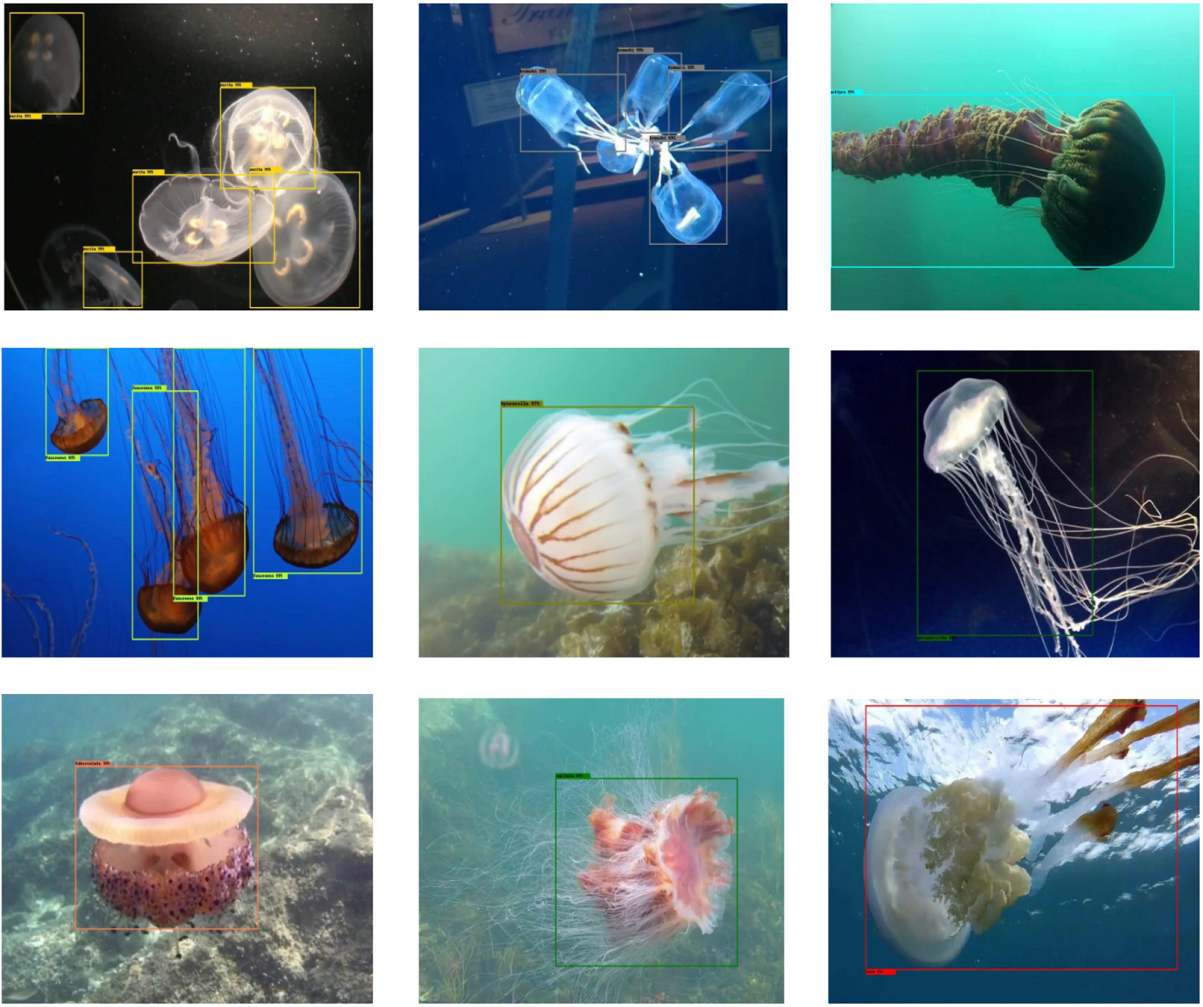
Jellyfish detection examples. From left to right and top to bottom: *A. aurita*, *C. branchi*, *C. achlyos*, *C. fuscescens*, *C. hysoscella*, *C. quinquecirrha*, *C. tuberculata*, *C. capillata*, and *R. luteum*. Some of the pictures illustrated here show jellyfish in aquaria environment; however, most of the pictures used to train the network were taken in natural environments.

As a first step, a set of images containing jellyfish is used as input for a frozen version of a deep object detection neural network‐trained model. During inference, the network starts the process of jellyfish detection. Subsequently, detection is optimized by a nonmaxima suppression (nms) algorithm (Neubeck & Van Gool, 2006). The final predictions are obtained by deleting instances with an associated confidence lower than a selected threshold value (Cthr). Figure 2 shows a representation of identification bounding boxes for several of the jellyfish species.

### 2.5 Evaluation metrics

To evaluate the performance of a trained model, the bounding‐box predictions generated as a result of the test dataset are classified as either a true positive, TP; or a false positive, FP. To do so, we use the Intersection over Union (IoU) metric. IoU is defined as the area of the intersection between a predicted and a ground‐truth bounding box, divided by the union area of these bounding boxes (Equation 1).

(1) $$\mathrm{IoU}=\frac{A_{\text{intersection}}}{A_{\text{union}}}$$

A prediction is classified as TP if the IoU value with any ground‐truth bounding box is greater than a threshold IoU and the predicted species class (i.e., species of interest) matches the class specified in the ground‐truth box. When these conditions are not met, the prediction is classified as a FP (Equation 2). Ground‐truth instances that do not have an IoU greater than the threshold IoU with any prediction are considered as undetected instances (false negatives, FN). Following the criteria applied in the PASCAL VOC challenge (Everingham et al., 2010), the IoU threshold was set at 0.5 (thr_iou_ = 0.5).

(2) $$\begin{array}{c} \text{Prediction}=\left\{\begin{array}{c} \mathrm{TP},\text{if}\;\mathrm{IoU}\geq {\mathrm{thr}}_{\mathrm{iou}}\&{\text{Class}}_{\text{pred}}={\text{Class}}_{\mathrm{gt}}\\ \mathrm{FP},\text{otherwise} \end{array}\right. \end{array}$$

Once the number of TP, FP and FN is obtained, the average precision (AP) is calculated (Zhu, 2004); AP is one the most frequently used metrics in object detection applications. This metric is defined as the area under the max (precision)‐recall curve. Once the AP is obtained for each class, a mean average precision (mAP) for all classes is computed. In addition, the confidence score generated by the network for each prediction can be used to fine‐tune the network. The confidence score is used to find a confidence value that, when deleting predictions with low confidence values, more FP than TP are deleted thus, improving the detection performance of the network. To do this, a threshold sweep on the confidence prediction from 0% to 100% was performed in 1% steps (C_thr). For each step, the predictions with an associated confidence level lower than the C_thr were removed; and the Precision, Recall, and F1‐score metrics were calculated from the TP, FP, and FN values.

Precision is calculated as the percentage between correctly identified instances and all the predictions made by the model (Equation 3). Recall refers to the percentage of TP detections with respect to all jellyfish instances present on the test data (Equation 4). Finally, the F1‐score combines the Precision and Recall metrics (Equation 5).

(3) $$\text{Precision}=\frac{\mathrm{TP}}{\mathrm{TP}+F}\times 100$$

(4) $$\text{Recall}=\frac{\mathrm{TP}}{\mathrm{TP}+\mathrm{FN}}\times 100$$

(5) $$F1‐\text{score}=2\times \frac{\text{Recall}\times \text{Precision}}{\text{Recall}+\text{Precision}}\times 100$$

Furthermore, a multiclass confusion matrix is generated using all classes TP and FP counts. A multiclass confusion matrix is a *N* × *N* matrix, where *N* is the number of studied classes. In our case, each row of the matrix corresponds to a jellyfish species and indicates, in the matrix columns, the percentage of the detections made by the neural network that correspond to each species of jellyfish.

For each of the four models corresponding to the different geographical areas, a 5‐k‐fold validation was performed (Geisser, 1975). Through this method, the dataset is split into five equally sized subsets and the network is trained five times, each time using a different subset as the test data (20% of the dataset) and the remaining four subsets as training data (80% of the dataset). The final results (i.e., AP, mAP, C_thr, Recall, Precision, and F1‐score) are thus an average of the five trainings. This method reduces the variability of the results, making them less dependent on the selected test and training sets and therefore obtaining a more accurate performance estimation.

## 3 RESULTS

For each one of the four geographical regions, the AP, mAP, Cthr, and F1‐score metrics are presented, along with a multiclass confusion matrix. The confusion matrix allows us to identify those pairs of classes where the network has difficulties in telling them apart.

### 3.1 Atlantic–Mediterranean region

**FIGURE 3:**
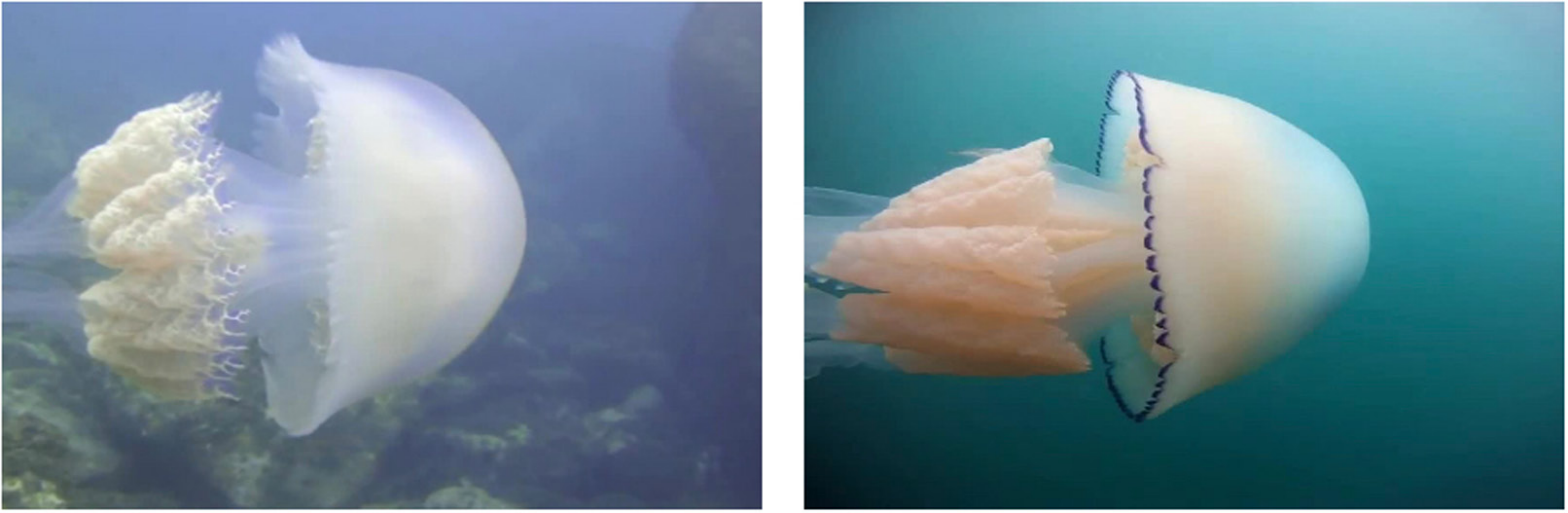
Comparison between the *R. luteum* (left) and *R. pulmo* (right) jellyfish species.

The spatial distribution assessment indicated that 12 of the 15 jellyfish species had been recorded in the Atlantic/Mediterranean area. The AP values for approximately 60% of those species ranged between 90% and 99%. The rest of the species, that is, *A. aurita*, *C. branchii*, *R. luteum*, and *P. noctiluca* presented values ranging between 75% and 80%. The mAP for all classes was 87.%. After performing the confidence threshold sweep, an F1‐score of 83.9% was obtained when applying a confidence threshold of 67.4% (Table 2). The multiclass confusion matrix indicates that the network does not tend to get confused between species. The exception was the capacity of the network to distinguish between *R. pulmo* and *R. luteum* as the network confused them in 17.9% of the cases. Two main reasons are attributable to this weakness: on the one hand, both species belong to the same genus and are very similarly looking (Figure 3). On the other hand, the training data for *R. pulmo* were thrice that of *R. luteum*, biasing the network toward *R. pulmo* (Table 3).

**TABLE 2:** Performance metrics for the Atlantic/Mediterranean regional model

Species	AP	mAP	C_THR	REC	PREC	F1‐SCORE
*A. aurita*	81.2%					
*C. branchi*	77.3%					
*C. hysoscella*	79.6%					
*C. quinquecirrha*	91.3%					
*C. tuberculata*	91.1%					
*C. capillata*	94.7%	87.7%	67.4%	80.1%	88.0%	83.9%
*C. lamarckii*	93.5%					
*P. noctiluca*	74.6%					
*R. luteum*	79.8%					
*R. pulmo*	96.9%					
*S. meleagris*	93.1%					
*T. ohboya*	98.9%					

Abbreviations: AP, average precision; C_THR, confidence threshold; mAP, mean average precision; PREC, precision; REC, recall.

**TABLE 3:** Multiclass confusion matrix for the species recorded in the Atlantic/Mediterranean region

Species	*A. aurita*	*C. branchi*	*C. hysoscella*	*C. quinquecirrha*	*C. tuberculata*	*C. capillata*	*C. lamarckii*	*P. noctiluca*	*R. luteum*	*R. pulmo*	*S. meleagris*	*T. ohboya*
*A. aurita*	99.5%	0.1%	‐	‐	‐	‐	‐	‐	‐	0.1%	0.3%	‐
*C. branchi*	2.4%	93.5%	‐	‐	‐	‐	‐	‐	‐	‐	‐	4.1%
*C. hysoscella*	‐	‐	93.8%	‐	0.3%	0.3%	‐	5.2%	‐	0.3%	‐	‐
*C. quinquecirrha*	‐	‐	‐	95.9%	‐	‐	‐	4.1%	‐	‐	‐	‐
*C. tuberculata*	‐	‐	0.9%	‐	95.3%	‐	‐	0.6%	‐	1.8%	1.5%	‐
*C. capillata*	‐	‐	‐	‐	0.5%	97.4%	1.5%	‐	‐	0.5%	‐	‐
*C. lamarckii*	‐	‐	‐	‐	‐	3.3%	95.1%	‐	‐	1.6%	‐	‐
*P. noctiluca*	0.3%	‐	‐	‐	‐	‐	‐	99.4%	‐	0.3%	‐	‐
*R. luteum*	‐	‐	‐	‐	‐	‐	‐	‐	81.1%	17.9%	‐	0.9%
*R. pulmo*	0.3%	‐	‐	‐	‐	‐	‐	0.3%	0.3%	98.6%	0.3%	‐
*S. meleagris*	‐	‐	‐	‐	‐	‐	‐	‐	‐	3.9%	96.1%	‐
*T. ohboya*	‐	‐	‐	‐	‐	‐	‐	0.4%	‐	‐	‐	99.6%

### 3.2 Pacific region

Seven of the 15 jellyfish species had been detected in the Pacific area. The network model for this region had a high performance as the AP values for the majority of the species laid between 90% and 99%, while only two species (*P. noctiluca* and *A. aurita*) had values comprised between 78% and 83%. The mAP had a value of 90.1% and a F1‐score of 83.5% after applying an optimal confidence threshold of 44.8% (Table 4). The multiclass confusion matrix indicates that the network performed very well in distinguishing between species, as all values but one were above 98% with the exception of *N. nomurai* which was correctly identified in 96.7% of the cases. Only occasionally (3.3% of the cases) was misidentified as *C. capillata* (Table 5).

**TABLE 4:** Performance metrics for the Pacific regional model

Species	AP	mAP	C_THR	REC	PREC	F1‐SCORE
*A. aurita*	82.5%					
*C. achlyos*	99.3%					
*C. fuscescens*	83.4%					
*C. capillata*	99.5%	90.1%	44.8%	82.2%	84.9%	83.5%
*N. nomurai*	91.6%					
*P. noctiluca*	78.1%					
*S. meleagris*	96.0%					

Abbreviations: AP, average precision; C_THR, confidence threshold; mAP, mean average precision; PREC, precision; REC, recall.

**TABLE 5:** Multiclass confusion matrix for the species recorded in the Pacific region

Species	*A. aurita*	*C. achlyos*	*C. fuscescens*	*C. capillata*	*N. nomurai*	*P. noctiluca*	*S. meleagris*
*A. aurita*	100.0%	‐	‐	‐	‐	‐	‐
*C. achlyos*	‐	100.0%	‐	‐	‐	‐	‐
*C. fuscescens*	‐	‐	99.6%	‐	‐	‐	0.4%
*C. capillata*	‐	0.5%	‐	99.5%	‐	‐	‐
*N. nomurai*	‐	‐	‐	3.3%	96.7%	‐	‐
*P. noctiluca*	‐	‐	‐	‐	‐	100.0%	
*S. meleagris*	‐	‐	0.8%	0.8%	‐	‐	98.4%

### 3.3 Arctic–Baltic region

Four of the 15 species were recorded in the Arctic/Baltic region. All AP values for this region were above 80%. The values for *C. capillata* and *C. lamarckii* were both over 97%, while the AP values for *A. aurita* and *C. fuscescens* were in the lower eighties (81.5% and 83.2%). The average AP between all the species was slightly over 90%. After applying the optimal confidence threshold of 55.4%, the F1‐score achieved a value of 82.2% (Table 6). The performance of the network model was very high as it was able to distinguish between the different species. Even very similarly looking species belonging to the same genus (i.e., *C. capillata* and *C. lamarckii*) were correctly identified in 98% of the cases (Table 7).

**TABLE 6:** Performance metrics for the Arctic/Baltic regional model

Species	AP	mAP	C_THR	REC	PREC	F1‐SCORE
*A. aurita*	81.5%					
*C. fuscescens* *C. capillata*	83.2% 97.4%	90.1%	55.4%	80.7%	83.7%	82.2%
*C. lamarckii*	98.4%					

Abbreviations: AP, average precision; C_THR, confidence threshold; mAP, mean average precision; PREC, precision; REC, recall.

**TABLE 7:** Multiclass confusion matrix for the species recorded in the Arctic/Baltic region

Species	*A. aurita*	*C. fuscescens*	*C. capillata*	*C. lamarckii*
*A. aurita*	100.0%	‐	‐	‐
*C. fuscescens*	0.4%	99.6%	‐	‐
*C. capillata*	‐	0.5%	98.0%	1.5%
*C. lamarckii*	‐	0.0%	1.6%	98.4%

### 3.4 Indian Ocean–South China Sea Region

The model for the Indian Ocean and the South China Sea was developed with the four jellyfish species recorded in the region. The AP values for two of the species, *N. nomurai* and *C. quinquecirrha*, were very high, ranging between 96.7% and 97.5%. AP values for *A. aurita* and *P. noctiluca* were slightly lower, 82.4% and 79.2%, respectively. The mAP had a value of 89%, while the F1‐score was 80.8% after applying a 64.8% confidence threshold (Table 8). As with the other models, the network had a high performance in distinguishing between species, as all values were above 99.5% (Table 9).

**TABLE 8:** Performance metrics for the Indian Ocean/South China Sea regional model

Species	AP	mAP	C_THR	REC	PREC	F1‐SCORE
*A. aurita*	82.4%					
*C. quinquecirrha* *N. nomurai*	96.9% 97.5%	89.0%	64.8%	77.5%	84.4%	80.8%
*P. noctiluca*	79.2%					

Abbreviations: AP, average precision; C_THR, confidence threshold; mAP, mean average precision; PREC, precision; REC, recall.

**TABLE 9:** Multiclass confusion matrix for the species recorded in the Indian Ocean/South China Sea region

Species	*A. aurita*	*C. quinquecirrha*	*N. nomurai*	*P. noctiluca*
*A. aurita*	99.9%	‐	0.1%	‐
*C. quinquecirrha*	‐	100.0%	‐	‐
*N. nomurai*	‐	‐	100.0%	‐
*P. noctiluca*	‐	0.5%	‐	99.5%

Overall, the performance metrics are good and consistent over the four regions. Furthermore, the network does not tend to mistake a jellyfish species for a wrong one, demonstrating the effectiveness of the implemented strategy of dividing the species into four regions.

## 4 DISCUSSION

Thus far, jellyfish have proven difficult to monitor, due to the high associated cost of logistics and the need of humans to analyze visual data (direct observations or video recordings), limiting both the spatial and temporal scales of monitoring programs (but see Condon et al., 2015). The results of this paper demonstrate that the application of CNNs to image data has the potential to deliver a tool that enables the cost‐effective collection of jellyfish data on larger spatial and temporal scales. In our initial publication, we described the successful automatic classification and quantification of three jellyfish species common in the Mediterranean (Martin‐Abadal et al., 2020). The results were highly promising, but the question remained of whether the CNN would be able to distinguish a much larger number of species and thus serve as a backbone for the development of a global tool. Here, we demonstrate that the algorithms developed are robust and that most species can be distinguished by the system with high precision and an acceptable level of confusion. The tool in its original unmodified version showed weaknesses when applied on a larger number of species and a restructuring of the approach architecture was necessary to achieve acceptable levels of error in the classifications. Four separate world subareas models were developed to avoid confusion between similarly looking species, which did not overlap in their geographical distributions. Through this modification, errors in misidentifications were reduced; nevertheless, some species confusions persisted. We note that in our attempt to construct a global CNN, we expected the performance of the CNN to improve with increasing number of images. However, when increasing image numbers for the global CNN, precision did not increase as would be expected. While the overall number of images was higher, images for each individual species might not have been sufficient. When the CNN precision does not improve sufficiently with image number to run at such a scale, the networks can be divided into submodels, corresponding to different geographical areas. This was our approach. The development of the CNN as a single model would be ideal but is only justified when increasing the number of images improves the CNN. We expect the performance of the CNN to improve with increasing numbers of images for each species. In the event that the performance of the CNN does not improve sufficiently to run as a single model with worldwide application, the network can still be left divided into the existing (or modified) submodels, corresponding to different geographical areas. The development of the CNN as a single model would be the ideal objective but the aim is to have a system that is able to facilitate the identification of jellyfish species, which is still achieved through the CNN working as separate submodels.

Within the Atlantic–Mediterranean subarea model, the most notable case was for *Rhizostoma pulmo* and *Rhizostoma luteum* as the network confused them in 17.9% of the cases. This may be related to the very similar morphology of the two species or to the fact that for *R. pulmo* we had thrice the amount of training data available, making the network more biased toward this species. In the other subarea models, confusion percentages were much lower (max. 3.3%). Overall, the average precision values (APs) for most of the species varied between 90% and 99%. However, there were some species, such as *A. aurita*, *C. branchi*, *R. luteum*, or *P. noctiluca*, that showed lower AP values (75%–80%). These lower AP values may be related to multiple factors, ranging from insufficient training data to high similarities in body morphology between different species. For instance, *P. noctiluca* is a translucent organism whose body can adopt significantly different configurations, due to the movement of their tentacles; or in the case of *A. aurita*, it tends to appear in big blooms, causing overlapping in the images and making it hard to identify single organisms. While the classification weaknesses for some species may be improved with the integration of additional training data, for others there may be morphological or behavioral aspects that will make it difficult to increase the overall classification precision. Nevertheless, in general, the incorporation of additional training data is expected to significantly improve the detection and correct classification of objects (Goodfellow et al., 2016).

The comparison of the mean average precision values of Jellytoring 2.0 with other studies dealing with a similar number of species (i.e., 10), shows that Jellytoring 2.0 outperforms previous attempts. While the mAP Jellytoring 2.0 ranged from 88% to 90%, Han et al. (2021) showed an mAP of 75%. By contrast, when compared to studies that automatically identified a lower number of species (i.e., 5), Jellytoring 2.0 performance was lower, Gauci et al. (2020) mAP = 96%. However, algorithms trained to identify a lower number of jellyfish species tend to perform better, as shown with the original version of Jellytoring (mAP = 95%).

**FIGURE 4:**
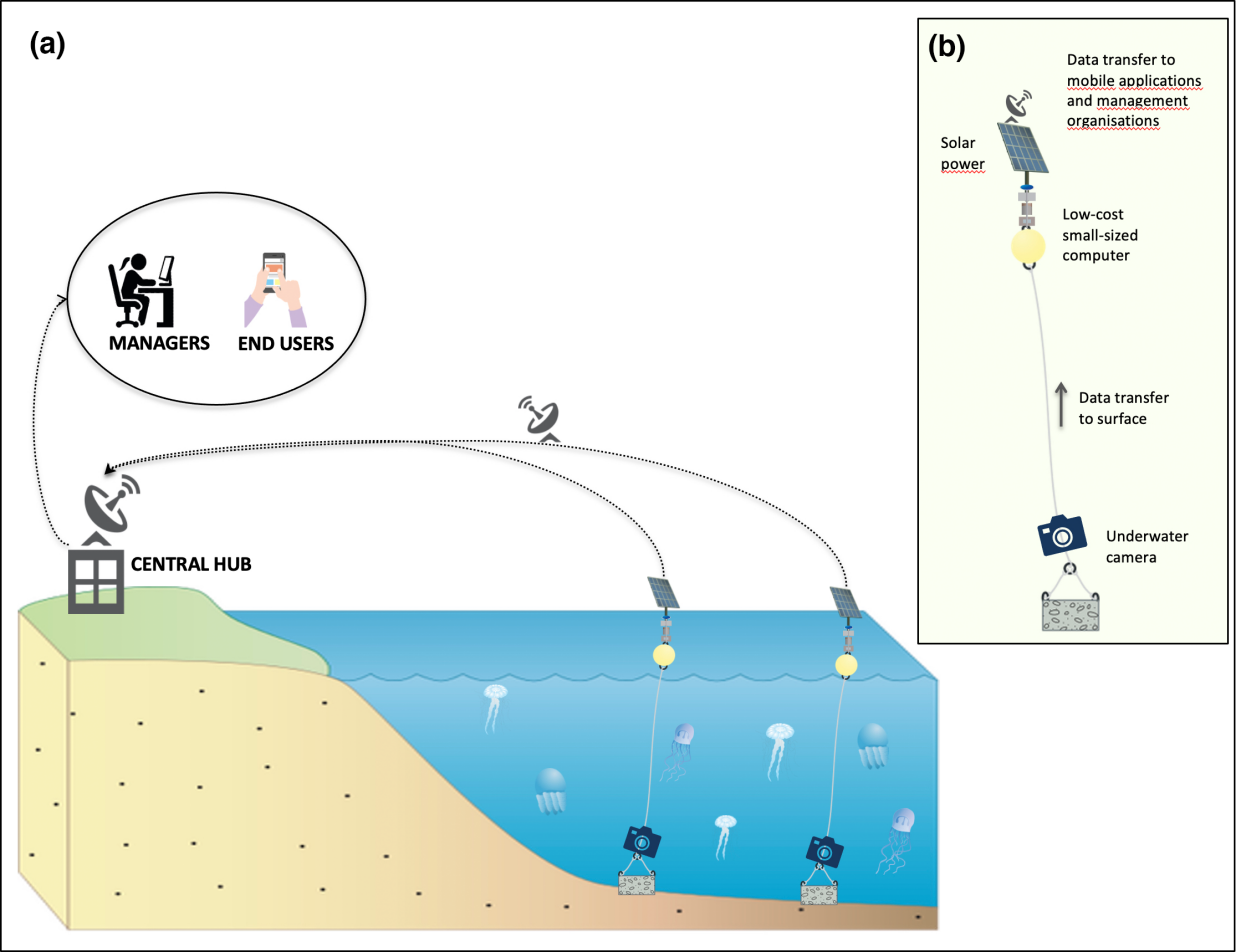
(a) Conceptual diagram for a real‐time jellyfish monitoring system. (b) Detailed diagram of a jellyfish monitoring camera system with integrated CNN.

With this paper, we want to offer the scientific and nonscientific community the basis for an automatic jellyfish identification system that could form the first step toward the creation of large‐scale and long‐term monitoring datasets, as well as real‐time monitoring tools. Although not included as part of this exercise due to insufficient image data for each of the species considered here, Jellytoring is capable of performing real‐time quantification of the identified jellyfish species (Martin‐Abadal et al., 2020). This feature allows for the development of real‐time monitoring tools in coastal management scenarios. The rapid development of cheap hardware video‐capturing devices and software processing units allows for the integration of CNNs to generate in situ identification and abundance data. Data captured and generated through these assemblages can subsequently be sent via communication systems to land‐based hubs where data can be further processed and visualized in near real‐time (Figure 4). Such monitoring systems can have a wide range of applications as early warning systems for commercial operation and coastal management and can also provide invaluable data for science helping to close some of the remaining gaps in jellyfish ecology.

It is with this in mind and to extend the scope of the Jellytoring 2.0 tool that we ask the scientific community to actively participate in the provision of image data for both new species not yet included in the application as well as for species already covered. To facilitate the provisioning of this data we have created a web application (jellytoring.uib.es, under development) that will allow uploading photos and video files, as well as the positioning of bounding boxes and classification labels. For the effective and accurate performance of the tool, large numbers of training images are required, especially if the tool is to become a global application. While currently our website only accepts the uploading of still images, still images from videos can be easily extracted by the user using dedicated free software such as MPEG. This type of software is capable of rapidly converting entire video clips to images, or to extract still frames in a time‐frequency set by the user. The results provided by Jellytoring 2.0 will be in form of a data file that will show the time and video frame an individual object was detected, its classification by the network and a confidence and confusion indicator of that classification. From this raw data, the user will be able to easily calculate various derived parameters such as overall abundances within the video footage, abundances per time interval or diversity. The results of this study have been very encouraging but only with the involvement of the wider scientific community and interested citizens can this application grow into a global application.

## AUTHOR CONTRIBUTIONS

**Ana Ruiz‐Frau:** Conceptualization (lead); data curation (lead); formal analysis (lead); funding acquisition (lead); investigation (lead); methodology (equal); project administration (lead); resources (lead); software (supporting); supervision (lead); validation (supporting); visualization (equal); writing – original draft (lead); writing – review and editing (lead). **Miguel Martin‐Abadal:** Conceptualization (supporting); data curation (lead); formal analysis (lead); funding acquisition (supporting); investigation (supporting); methodology (lead); project administration (supporting); resources (supporting); software (lead); supervision (equal); validation (equal); visualization (lead); writing – original draft (equal); writing – review and editing (equal). **Charlotte Louise Jennings:** Conceptualization (supporting); data curation (equal); formal analysis (equal); funding acquisition (supporting); investigation (supporting); methodology (supporting); project administration (supporting); software (supporting); supervision (supporting); validation (supporting); visualization (supporting); writing – original draft (supporting); writing – review and editing (supporting). **Yolanda Gonzalez‐Cid:** Conceptualization (supporting); data curation (supporting); formal analysis (lead); funding acquisition (supporting); investigation (equal); methodology (equal); project administration (supporting); resources (equal); software (equal); supervision (equal); validation (equal); visualization (equal); writing – original draft (equal); writing – review and editing (equal). **Hilmar Hinz:** Conceptualization (lead); data curation (equal); formal analysis (equal); funding acquisition (supporting); investigation (equal); methodology (equal); project administration (supporting); resources (supporting); software (supporting); supervision (equal); validation (equal); visualization (equal); writing – original draft (lead); writing – review and editing (lead).

## Supporting information


Table S1

Table S2

## Data Availability

The dataset and code used is deposited in a GitHub repository https://github.com/srv/jf_object_detection/tree/regions.
