# Corrigendum

**DOI:** 10.1002/ece3.9575

**Published:** 2022-11-27

In the recent article by Ruiz‐Frau et al. (2022), the correct affiliation of Charlotte L. Jennings is Institut Mediterrani d'Estudis Avançats, IMEDEA (CSIC‐UIB), Esporles, Spain.

The authors apologize for the error.
