# Supplementary material for: The potential of Jellytoring 2.0 smart tool as a global jellyfish monitoring platform
Source: Ecol Evol. 2022 Nov 1;12(11):e9472. doi: 10.1002/ece3.9472 (PMC9627081; doi:10.1002/ece3.9472)
Supplement: Supplementary file 1 — Table S1 [file ECE3-12-e9472-s002.docx]

**Table SI 1.** Performance metrics for the model considering all jellyfish species (AP = average precision, mAP= mean average precision, C_THR = confidence threshold, REC = recall, PREC = Precision)

| Species | AP | mAP | C_THR | REC | PREC | F1-SCORE |
| --- | --- | --- | --- | --- | --- | --- |
| *A. aurita* | 77,1% |  |  |  |  |  |
| *C. branchi* | 63,7% |  |  |  |  |  |
| *C. achlyos* | 99,4% |  |  |  |  |  |
| *C. fuscescens* | 84,3% |  |  |  |  |  |
| *C. hysoscella* | 70,8% |  |  |  |  |  |
| *C. quinquecirrha*  *C. tuberculata* | 99,4%  93,3% | 84,1% | 17% | 82,2% | 82,6% | 82,4% |
| *C. capillata* | 77,7% |  |  |  |  |  |
| *C. lamarckii* | 95,4% |  |  |  |  |  |
| *N. nomurai* | 85,7% |  |  |  |  |  |
| *P. noctiluca* | 75,6% |  |  |  |  |  |
| *R. luteum* | 70,4% |  |  |  |  |  |
| *R. pulmo* | 94,6% |  |  |  |  |  |
| *S. meleagris* | 73,8% |  |  |  |  |  |
| *T. ohboya* | 99,9% |  |  |  |  |  |
