# Supplementary material for: The potential of Jellytoring 2.0 smart tool as a global jellyfish monitoring platform
Source: Ecol Evol. 2022 Nov 1;12(11):e9472. doi: 10.1002/ece3.9472 (PMC9627081; doi:10.1002/ece3.9472)
Supplement: Supplementary file 2 — Table S2 [file ECE3-12-e9472-s001.docx]

**Table SI 2.** Multi-class confusion matrix for all species

| Species | *A. aurita* | *C. branchi* | *C. branchi* | *C. fuscescens* | *C. hysoscella* | *C. quinquecirrha* | *C. tuberculata* | *C. capillata* | *C. lamarckii* | *N. nomurai* | *P. noctiluca* | *R. luteum* | *R. pulmo* | *S. meleagris* | *T. ohboya* |
| --- | --- | --- | --- | --- | --- | --- | --- | --- | --- | --- | --- | --- | --- | --- | --- |
| *A. aurita* | 97,0% | - | - | - | - | - | - | - | - | - | - | - | 3,0% | - | - |
| *C. branchi* | - | 88,0% | - | - | - | 4,0% | - | - | - | - | - | - | - | - | 8,0% |
| *C. branchi* | - | - | 100,0% | - | - | - | - | - | - | - | - | - | - | - | - |
| *C. fuscescens* | - | - | - | 93,2% | - | - | 1,7% | - | - | - | 5,1% | - | - | - | - |
| *C. hysoscella* | - | - | - | - | 83,1% | - | 7,7% | - | - | - | 7,7% | - | 1,5% | - | - |
| *C. quinquecirrha* | - | - | - | - | - | 100,0% | - | - | - | - | - | - | - | - | - |
| *C. tuberculata* | - | - | - | - | - | - | 100,0% | - | - | - | - | - | - | - | - |
| *C. capillata* | - | - | - | - | - | - | 10,0% | 82,5% | 7,5% | - | - | - | - | - | - |
| *C. lamarckii* | - | - | - | - | - | - | - | 4,0% | 96,0% | - | - | - | - | - | - |
| *N. nomurai* | - | - | - | - | - | - | 7,1% | - | - | 92,9% | - | - | - | - | - |
| *P. noctiluca* | - | - | - | 1,6% | - | - | - | - | - | - | 98,4% | - | - | - | - |
| *R. luteum* | - | - | - | - | - | - | - | - | - | - | - | 70,4% | 29,6% | - | - |
| *R. pulmo* | 1,7% | - | - | - | - | - | - | - | - | - | - | - | 98,3% | - | - |
| *S. meleagris* | - | - | - | - | - | - | 20,7% | - | - | - | - | - | 3,4% | 75,9% | - |
| *T. ohboya* | - | - | - | - | - | - | - | - | - | - | - | - | - | - | 100,0% |
